# Determination of p53 biomarker with a smart electrochemical biosensor based on brush polymer-functionalized disposable electrode

**DOI:** 10.55730/1300-0527.3736

**Published:** 2025-04-04

**Authors:** Muhammet AYDIN

**Affiliations:** Scientific and Technological Research Center, Tekirdağ Namık Kemal University, Tekirdağ, Turkiye

**Keywords:** Graft polymer, p53, impedimetric biosensor, electrochemical analysis

## Abstract

A label-less impedimetric biosensor modified with poly(thiophene)-graft-poly(glycidyl methacrylate) polymer (PThi-g-PGM) was manufactured for p53 protein quantification in human serum samples. In this study, PThi-g-PGM polymer was synthesized, and this polymer matrix was coated using the spin-coating technique on the single-use indium tin oxide (ITO) substrate for anti-p53 antibody immobilization. The anti-p53 antibodies with high affinity for p53 proteins were covalently attached onto the PThi-g-PGM-coated electrode surface. In addition, the affinity of anti-p53 for the p53 protein was monitored at a constant frequency. Under optimized conditions, the impedimetric changes were linearly related to the p53 concentrations, ranging from 0.05 to 15 pg/mL with a low detection limit of 15.9 fg/mL. This biosensor had desirable storage stability, acceptable repeatability, and high reproducibility. Moreover, this impedimetric biosensor could be regenerated through an acidic treatment procedure. Additionally, the suggested biosensor successfully detected the p53 antigen in human serum samples, and good recycling rates (98.41%–109.32%) were found. In summary, the proposed immunosensor may be a powerful tool for the analysis of the p53 protein for early detection of cancer biomarkers.

## 1. Introduction

One of the main causes of sickness and death worldwide is cancer, and the number of new cases is rapidly increasing [1,2]. Tumor markers are chemicals linked to cancer, the concentrations of which in bodily fluids or tissues may offer crucial data for clinical screening and early cancer detection. Because of this, developing reliable and precise tumor biomarker identification techniques early in the course of the disease is crucial [3,4]. Protein p53 plays a crucial part in cellular operation, including cell proliferation regulation, DNA repair, and apoptosis [5]. Protein p53 acts as a tumor suppressor in cells [6]. Furthermore, the protein p53 is in charge of regulating cell reproduction and apoptosis in response to DNA damage. That is why selective recognition and analysis of p53 at very low concentrations can be used for cancer diagnosis at an early stage, and this improves treatment outcomes [7]. To quantify p53 levels, different techniques, such as high-performance liquid chromatography (HPLC), surface plasmon resonance (SPR), Raman spectroscopy, field-effect transistors, immunohistochemical techniques, and enzyme-linked immunosorbent assay (ELISA), have been utilized [8]. These analysis strategies have their own advantages, but there are obstacles, such as laborious procedures, low sensitivity, and inflexible designs. In addition, these techniques require skilled personnel. Therefore, easy, cost-effective, dependable, and highly sensitive techniques are required [9]. Zhou et al. developed a silver nanoparticle-modified SPR biosensor and utilized it for the analysis of p53 protein in humans with neck squamous cell cancer [10]. Song et al. fabricated a plasmonic biosensor for the investigation of the DNA–p53 protein, and this biosensor kinetically analyzed these interactions [11]. Amor-Gutiérrez et al. developed a competitive biosensor composed of a gold nanoparticle-coated screen-printed carbon electrode for the measurement of unfolded p53 protein. The biosensor showed a low limit of detection (LOD) (0.05 nM), and it was suitable for application in actual samples [12]. A fluorescent biosensor prepared with DNA-captured magnetic nanoparticles was constructed by Xu et al. for the monitoring of p53 protein expression. The analysis principle of this sensor was based on simple magnetic separation from the complex matrix, and it could analyze p53 protein from human cell lysate without extensive sample pretreatment [13].

Biosensors are small tools used to convert biological responses that can be applied in different fields, including biomarker measurement, food control, and environmental monitoring [14,15]. In most situations, electrochemical impedance spectroscopy (EIS) is employed as an electrochemical technique that provides information about molecular biointeractions [16,17]. This technique is suitable for analyzing the bulk and interfacial electrical properties and functions of biosensor systems [18].

In recent years, the production and application of conjugated polymers, particularly polythiophene, have gained interest due to their properties of facile production, good stability, and high conductivity. Additionally, these properties of polythiophene ensure proper conditions for the attachment of biological molecules [19,20]. The electrode modification with conjugated polymers containing functional groups is a feasible alternative for the immobilization of biomolecules. Thus, biomolecules can attach without requiring any crosslinkers [21]. For polymer layer coating on the working electrode, various techniques have been utilized. Spin-coating is one of them, and it produces reliable results on appropriate substrates. This method is simple, safe, and inexpensive, and it can produce homogenous and high-quality polymer films [22,23].

In this work, an ITO-based disposable biosensor was designed for label-free and sensitive analysis of the p53 cancer biomarker. The PThi-g-PGM polymer was employed as a matrix in the biosensor production process, and it exhibited appropriate biocompatibility and low toxicity for the loading of biomolecules. Because of the glycidyl side groups of the PThi-g-PGM polymer, anti-p53 bound directly onto the PThi-g-PGM-modified electrode without the need for a crosslinking agent. Anti-p53 antibody could specifically recognize its p53 antigen, and the specific biointeraction between anti-p53 and p53 antigen was utilized to detect the p53 antigen. Furthermore, the PThi-g-PGM polymer matrix material provided biomolecules with a large surface area and outstanding stability. For the fabrication of the electrode, a spin-coating technique was utilized, and it simplified and facilitated the preparation of the biosensor. Electrochemical and microscopic methods were used to characterize changes on the working electrode surface. Finally, the developed biosensor was utilized to analyze p53 in serum samples. In addition, the repeatability, reproducibility, and reusability of the sensor were also tested.

## 2. Experimental

### 2.1. Chemicals and materials

3-thiophene acetic acid, 1,3-dibromo-2-propanol, N-(3-dimethylaminopropyl)-N’-ethylcarbodiimide hydrochloride, 4-dimethylaminopyridine, anhydrous iron(III) chloride, glycidyl methacrylate, N,N,N’,N”,N”-pentamethyldiethylenetriamine, copper(II) bromide, tetrahydrofuran, anisole, magnesium sulfate, indium tin oxide sheet (0.5 cm × 2 cm, 60 Ω/cm^2^), potassium ferricyanide, potassium ferrocyanide, potassium chloride, acetone, disodium phosphate, and monosodium phosphate, monoclonal anti-p53 antibody produced in mouse, p53 human antigen, bovine serum albumin (BSA), interleukin-1β human (IL-1β), interleukin-8 human (IL-8), interleukin-1α human (IL-1α), interleukin-6 human (IL-6), and human serum samples were from Sigma-Aldrich (St. Louis, MO, USA).

### 2.2. Instrument conditions

All electrochemical studies were performed on a Gamry Reference 1000 (Warminster, PA, USA) electrochemical workstation with a three-electrode module: a platinum wire auxiliary electrode, an Ag/AgCl reference electrode, and an ITO working platform. The morphologies of the electrode surfaces were observed using a scanning electron microscope (SEM, Quanta FEG 250, FEI, Hillsboro, OR, USA) and atomic force microscope (AFM, AFM PLUS+, NanoMagnetic). SEM-EDX pictures were recorded using SEM. FTIR measurement was performed with a Bruker Corporation Vertex 70 spectrometer (Karlsruhe, Germany).

### 2.3. Electrochemical studies

All analyses were performed in a phosphate buffer containing 5 mM [Fe(CN)_6_]^3−/4−^ solution with a traditional three-electrode module. The EIS spectra were attained in the working frequency range from 0.05 Hz to 50 kHz. Cyclic voltammograms (CV) were recorded at a scan rate of 100 mV/s in the potential working range from −500 mV to 1000 mV. Single frequency impedance (SFI) measurement was recorded at a constant frequency of 8 Hz.

#### 2.3.1. Synthesis of glycidyl group-bearing polythiophene

The PThi-g-PGM polymer brushes were successfully prepared through a series of reactions (esterification, oxidative polymerization, and ATRP) (Figure S1). The thiophene compound was synthesized via an esterification reaction, which was used as a monomer for the thiophene macroinitiator [24]. The polythiophene macroinitiator was prepared via a chemical oxidation polymerization process [25]. The PThi-g-PGM polymer brushes were prepared via an atom transfer radical polymerization technique [2,26], and this procedure was consistent with our earlier report. FTIR (ATR, cm^−1^): 2995, 2938; 1722 (C=O); 1448; 1252; 1139; 905; 845 (C–O–C); 754; 537 (C–S–C); 452 (Figure S2). Raman (λ_laser_=780 nm): 3006, 2938; 1730 (C=O); 1481; 1450; 1258; 1135; 909, 852 (C–O–C); 760; 606; 535 (Figure S3).

### 2.4. Fabrication of p53 analysis platform

The electrochemical biosensing platform construction procedure is presented in Scheme. In brief, the ITO-coated polyethylene terephthalate (PET) was first sonicated with acetone, soap solution, and distilled water for 5 min each, respectively. Next, it was dried under inert nitrogen gas. The clean ITO substrate was placed onto the spin-coater platform. The immobilization matrix, PThi-g-PGM polymer, was dripped onto the ITO-PET substrate, and the platform was spin-coated for 1 min. Thus, a PThi-g-PGM film was prepared on the ITO substrate. The PThi-g-PGM-modified ITO-PET was then functionalized with anti-p53 antibodies by incubating it in anti-p53 solution. The anti-p53 antibody molecules bound to the working electrode surface through chemical covalent bonds. The antibody-immobilized electrode was rinsed with distilled water to remove any nonspecifically attached anti-p53 antibody. To avoid nonspecific attachment of anti-p53 antibody molecules to the coated ITO-PET electrode, the ITO/*PThi-g-PGM/*anti-p53 was incubated for 1 h in BSA solution, and then it was washed with distilled water. The final stage of the development process for the p53 biosensor, the ITO/PThi-g-PGM/anti-p53/BSA electrode, was incubated in a serially diluted target p53 antigen solution. After that, the modified ITO/PThi-g-PGM/anti-p53/BSA/p53 electrode was rinsed with distilled water. The electrochemical responses of the prepared surfaces at each step of modification were measured by EIS and CV techniques.

### 2.5. Surface characterization measurements

Topographical characterizations of the prepared electrodes were performed using SEM and AFM devices. The acceleration voltage and spot size of SEM analysis were 5 kV and 3.5 mm, respectively. Cantilevers with silicon probes were used in noncontact mode AFM. The AFM had a resolution of 256 pixels per line and a scan rate of 5 μm/s.

## 3. Results and discussion

The polymer synthesis and fabrication procedure of the manufactured immunosensor is illustrated in Scheme. In the polymer synthesis procedure, the grafting-from strategy was utilized because this strategy allows reliable control of the brush composition and structure.

In the initial stage of the manufacturing process, the immobilization platform of ITO/*PThi-g-PGM* was constructed using spin-coating of PThi-g-PGM polymer. The polymer film formation not only provided attachment points for anti-p53 antibodies but also offered a large surface area for antibodies (Step 1). In the second step, anti-p53 molecules bound covalently to the modified ITO platform surface (Step 2). In the third step, BSA molecules were used to block the anti-p53-immobilized electrode surface in order to stop the nonspecific attachment (Step 3). In the last stage of the procedure, the constructed platform surface was utilized to detect p53 antigen (Step 4).

### 3.1. Chemical features of modified ITO platform surfaces

**Figure 1:**
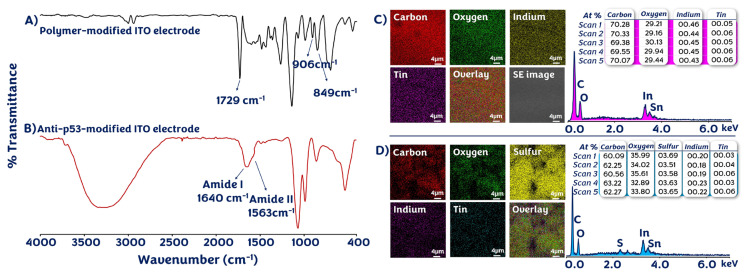
FTIR spectra of ITO/PThi-g-PGM (A) and ITO/PThi-g-PGM/anti-p53 electrodes (B) and EDX images of clean ITO (C) and ITO/PThi-g-PGM (D).

The FTIR spectra of modified electrodes further provided information about the successful fabrication of the biosensor. Figure 1A shows the FTIR spectra of ITO/PThi-g-PGM and ITO/PThi-g-PGM/anti-p53 electrodes. The spectrum of ITO/PThi-g-PGM had peaks at 849 and 906 cm^−1^ due to the glycidyl side groups of the PThi-g-PGM polymer [7]. Carbonyl group peaks of repeating units in the polymer were observed at 1729 cm^−1^ [27]. The spectrum of ITO/PThi-g-PGM/anti-p53 had broad and intense peaks for amide I and amide II at approximately 1640 cm^−1^ and approximately 1563 cm^−1^, respectively, due to the attachment of anti-p53 antibodies to the PThi-g-PGM polymer-coated electrode [28].

In addition, Figure 1B illustrates the SEM-EDX image of the clean ITO surface and the PThi-g-PGM brush polymer-coated ITO surface. According to EDX results, the clean surface contained oxygen, indium, and tin elements. After polymer film coating, the electrode surface contained the element sulfur (S) due to thiophene rings of PThi-g-PGM polymer, and this finding further confirmed that the polymer was deposited on the electrode surface. The percentage of sulfur in five distinct areas on the PThi-g-PGM-coated electrode surface was similar. These similar percentages demonstrated that the PThi-g-PGM polymer was uniformly coated on the electrode surface.

### 3.2. Electrochemical features of modified ITO platform surfaces

**Figure 2:**
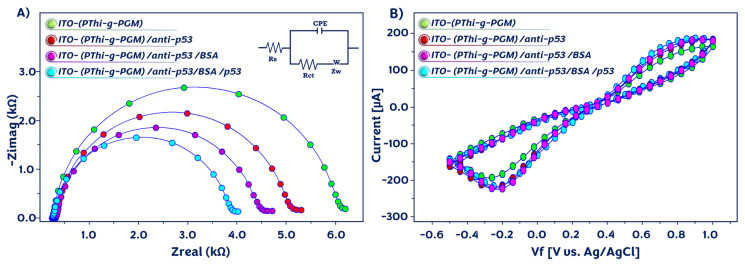
EIS and CVs of the different modified electrodes in a redox probe solution.

In order to monitor the changes in the impedimetric signal after each functionalization stage, EIS and CV techniques were employed. Nyquist plots (0.5 Hz–50 kHz) and CV (−0.5 to 1 V) were obtained in the ferri/ferro redox couple of 5 mM [Fe(CN)_6_]^3−/4−^. EIS was utilized as a convenient tool for monitoring interfacial features at the electrode surface. Figure 2A illustrates the Nyquist curves recorded during the fabrication procedure and the differences formed on the electrode surface. For accurate computation of R_ct_ for the differently coated electrode surfaces, a Randles equivalent circuit was employed to fit the Nyquist plots (Table S). Randles equivalent circuit contains four elements: a constant phase element (CPE), electrolyte solution resistance (R_s_), charge transfer resistance (R_ct_), and Warburg impedance (W) [23,29].

The PThi-g-PGM*-*coated electrode illustrated a small semicircle diameter owing to a fast electron transfer reaction, and a low ΔR_ct_ (3668 Ω) was measured. After immobilization of the anti-p53 antibody on the electrode surface, a notable rise in the semicircle diameter was observed (R_ct_ = 4160 Ω). In addition, the electrochemical impedance increased to 4774 Ω after surface blocking with BSA due to the blocking effect of the BSA protein. At the detection step, the attachment of p53 antigens to the modified ITO substrate surface resulted in an increase in the resistance (5878 Ω). Figure 2B illustrates the CV responses of the electrodes after each modification step. The ITO/PThi-g-PGM electrode exhibited a high electrochemical signal. When the electrode was coupled with anti-p53 antibodies, declines in peak currents were observed. The electrochemical signal declined notably because of the introduction of BSA biomolecules onto the ITO platform surface. After incubation with the p53 antigens, the peak currents decreased as a result of their attachment to the surface. As expected, the CV results confirmed the EIS results.

### 3.3. SEM and AFM characteristics of the p53 biosensor

**Figure 3:**
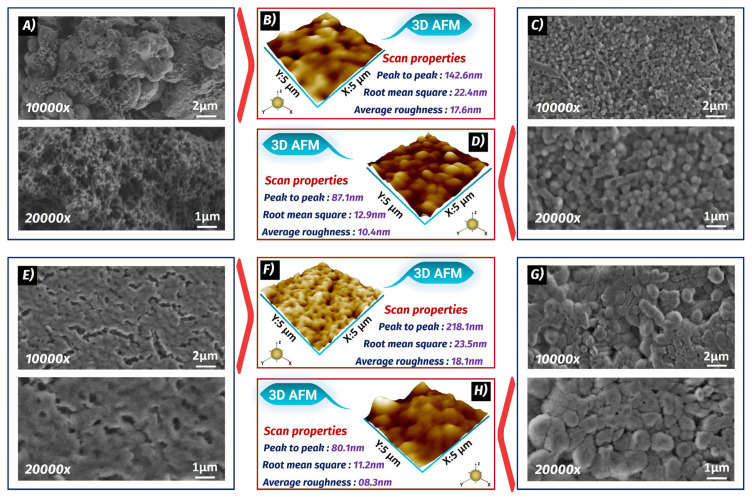
SEM and AFM micrographs of the different functionalized electrodes.

SEM and AFM monitoring techniques were utilized to examine the surface morphologies of the ITO platforms. As shown in Figure 3A, a classical poly(thiophene) polymer structure was observed on the working electrode surface, confirming its polymeric nature [30]. The average roughness (R_a_) of the PThi-g-PGM polymer-coated electrode was measured to be 17.6 nm (Figure 3B). In Figure 3C, globular structures were observed on the ITO substrate surface after the binding of anti-p53. The binding of anti-p53 molecules resulted in a smoother surface, and a low roughness average (R_a)_ was measured (Figure 3D).

Figure 3E illustrates the BSA-blocked immobilization platform, and as seen in this figure, BSA coated the free epoxy groups present on the anti-p53-modified substrate. After this step, the electrode morphology changed, and the R_a_ was found to be 18.1 nm (Figure 3F). In Figure 3G, a uniform binding of the p53 antigens to the immobilization platform was observed. This uniform binding resulted in a smooth surface, and a low R_a_ was obtained (8.3 nm, Figure 3H).

### 3.4. p53 analysis conditions optimization

**Figure 4:**
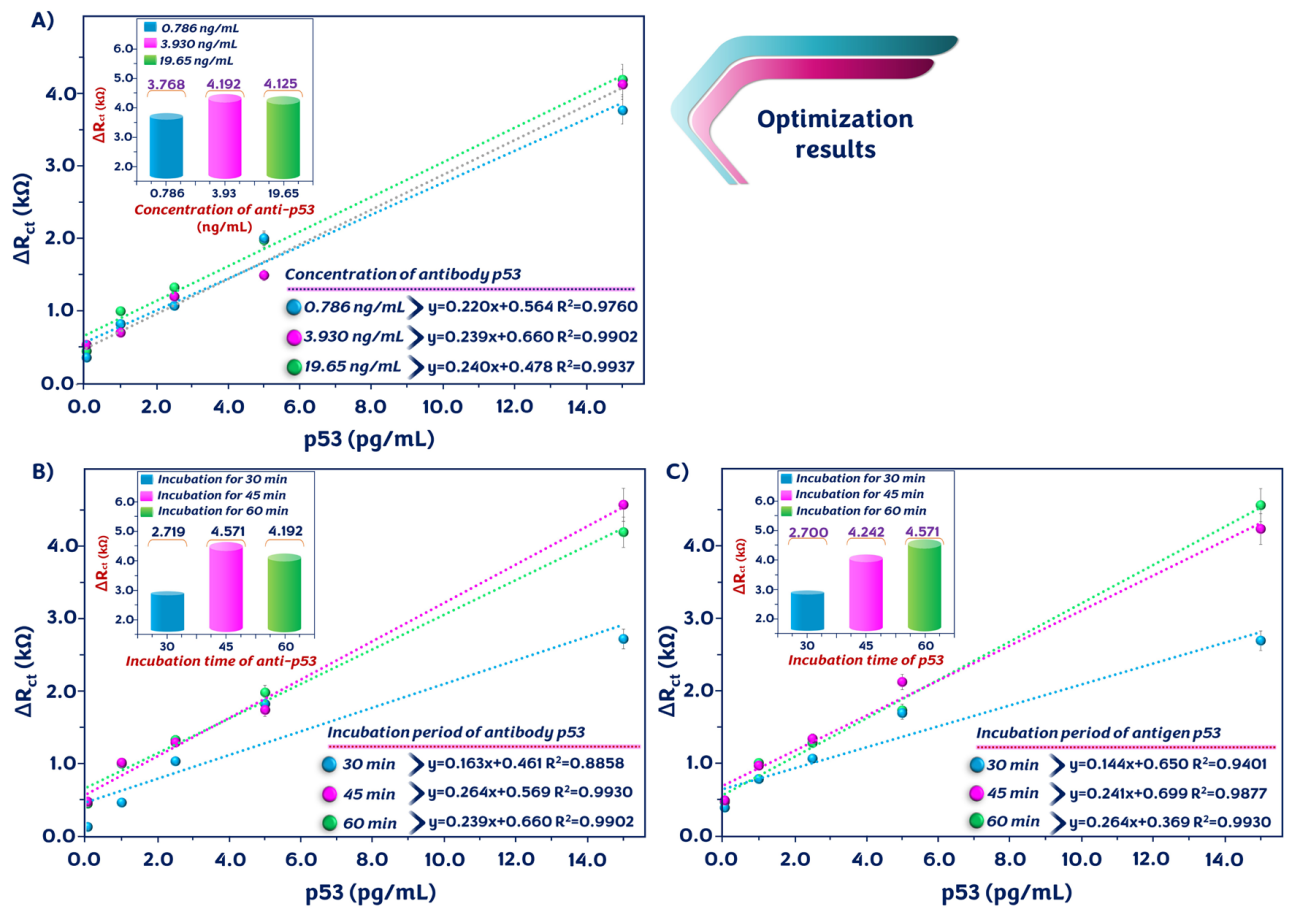
Biosensor responses to varying concentrations of anti-p53 antibodies (A) and to different times for anti-p53 (B), and p53 (C) proteins.

To improve the electrochemical performance of the immunosensor and obtain optimum experimental conditions, different parameters that influence the sensor response were optimized. One of these parameters was the concentration of anti-p53 antibody. Three different anti-p53 amounts (0.786, 3.93, and 19.65 ng/mL) were used, and the signals of biosensors prepared with these protein concentrations were measured. With increasing anti-p53 antibody levels, the impedimetric signals increased. The signals from the biosensor prepared with 3.93 ng/mL and 19.65 ng/mL were similar. Thus, 3.93 ng/mL was found to be the ideal concentration (Figure 4A).

The incubation time of the anti-p53 antibody, another important parameter, affected the impedimetric signal of the bioelectrode. The immunosensor had a low signal after 30 min of incubation. With increasing incubation times of anti-p53 antibodies, the impedimetric signal increased. As shown in Figure 4B, the maximum impedimetric response was recorded after 45 min of incubation. Another experimental parameter was the incubation time of the p53 antigen. The immunosensor showed an increasing electrochemical signal until the incubation time reached 60 min. Thus, 60 min was considered the optimal immobilization time (Figure 4C).

### 3.5. Analytical performance of the proposed system for p53 detection

**Figure 5:**
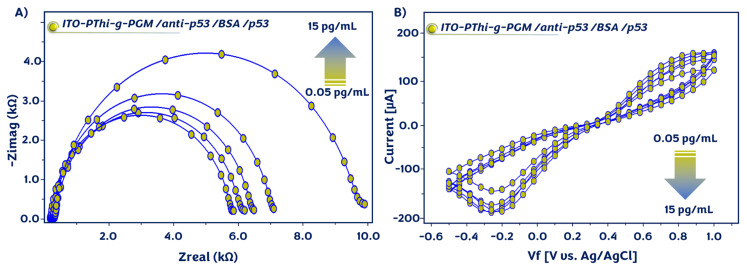
EIS (A) and CV (B) responses of the prepared system at increasing concentrations of p53.

**Figure 6:**
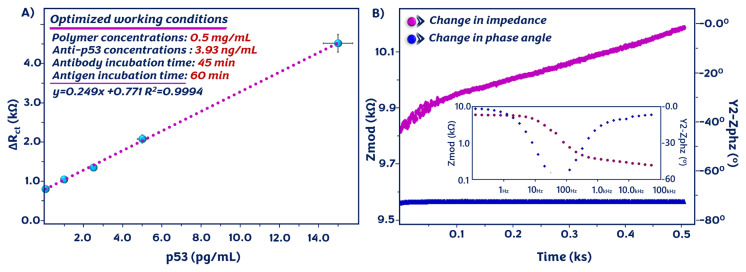
Linear relationship between the impedimetric response and p53 concentration (A), and impedance changes observed in the SFI analysis (B).

The recommended immunosensing system was applied to quantify the p53 antigen under optimal experimental conditions. The sensitivity of the suggested system was evaluated by EIS measurements, and the results are demonstrated in Figure 5A. The semicircle diameter increased continuously with the increasing p53 concentration. With the increase in p53 concentration, a higher amount of p53 interacted with p53 antibodies on the working electrode surface, resulting in greater antigen binding and a larger electrochemical response. In addition, the CV peak currents decreased continuously with the rising p53 level due to the formation of the electrostatic barrier. Thus, electron transmission between the ITO surface and the electrolyte solution became hindered (Figure 5B). A linear association between the p53 concentration and ΔR_ct_ (difference in charge transfer resistance before and after p53 immobilization) was found in the linear dynamic range of 0.05–15 pg/mL. The calibration plot shows that ΔR_ct_ was proportional to the p53 level (Figure 6A), and the linear regression equation was ΔR_ct_ = 0.249 [p53] + 0.771 (R^2^ = 0.9994). The LOD, quantification limit (LOQ), and sensitivity were computed as 15.9 fg/mL, 53.05 fg/mL, and 1.279 kΩ pg^−1^ mL cm^2^, respectively. The results indicated the great potential of the manufactured immunosensor for the quantitative detection of p53. The performance of the newly developed immunosensing system was compared to that of other p53 biosensors and ELISA kits (Table A). As investigated in Table A, the recommended immunosensor had a broad linear dynamic range, desirable sensitivity, and a low LOD. It was clearly observed that it could serve as a viable alternative to these commercial kits.

Apart from EIS and CV electrochemical analyses, an SFI experiment was performed to analyze the changes in impedance during immunoreaction. This technique is utilized for real-time monitoring of analytes. In this study, 8 Hz was used as a constant frequency (determined by the Bode plot, inset Figure 6B) during the SFI experiment. As shown in Figure 6B, the impedance of the system increased, indicating the capture of p53 antigens. Consequently, this technique can monitor the binding reaction in terms of both frequency and duration.

**Figure 7:**
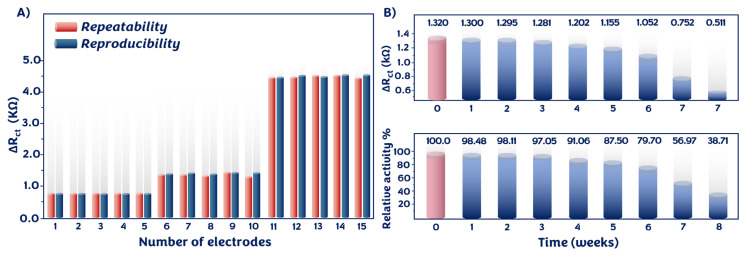
Results of repeatability and reproducibility (A) and storage-stability (B) tests.

The electrochemical signals of the p53 biosensor at concentrations of 0.05 pg/mL, 2.5 pg/mL, and 15 pg/mL were measured five times to evaluate the repeatability of the proposed system. The relative standard deviations (RSD) were computed to be 6.43%, 6.63%, and 0.95%, respectively, illustrating acceptable repeatability. The impedimetric responses of 15 distinct electrodes over several days were recorded to examine the reproducibility of the biosensor’s construction. The RSDs were calculated as 6.74%, 2.90%, and 0.86%. The low RSD values indicated good reproducibility of the fabricated system (Figure 7A).

Storage stability is an essential factor for a biosensor, and long-term stability with high activity demonstrates the success of the biosensor [31,32]. Therefore, this test was conducted as well. The time-dependent impedimetric responses of ten prepared immunosensors were tested weekly. The impedimetric response decreased slightly after 3 weeks of storage. The prepared immunosensor demonstrated good storage stability for 4 weeks, owing to strong covalent bonds between PThi-g-PGM and anti-p53 antibody (Ab). After storage at 4 °C for 6 weeks, the relative activity of the biosensor was 79.70% of its initial signal. These results indicated that the constructed biosensor would not be affected by the storage protocol (Figure 7B).

**Figure 8:**
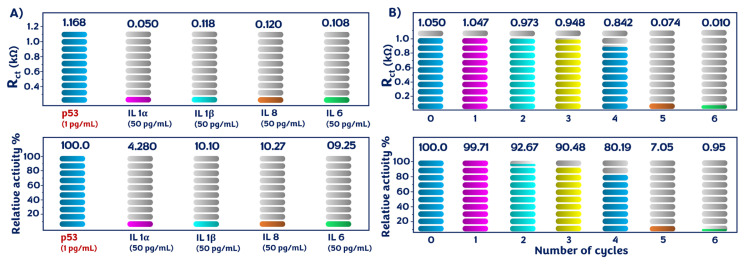
Selectivity (A) and regeneration (B) test results.

In addition to the storage-stability test, the biological selectivity of the fabricated system was assessed by examining various biomarker solutions: IL-1α, IL-1β, IL-8, IL-6, and p53. Compared to the results of p53, there was no discernible difference in the impedimetric response in the detection of interferent proteins. These results indicated that anti-p53 could not bind to the interfering proteins to generate signal responses. Only p53 interacted with anti-p53, and the results demonstrated the specific selectivity of the fabricated immunosensor (Figure 8A).

Regeneration of the electrode surface is important for the reusability of the biosensor [33]. In the regeneration process, the specific biointeraction between anti-p53 and p53 was disrupted using an acidic solution (0.1% HCl). After washing with ultrapure water, it was used for the measurement of the p53 antigen. The impedimetric responses of the immunosensor were recorded using the EIS technique after each regeneration cycle. After four regenerations with an acidic solution, the regenerated biosensor retained 80.19% of its initial response (Figure 8B). These results demonstrated the robust stability of the biosensor.

### 3.6. Serum sample analysis

The applicability of the prepared sensor to biological samples was also tested. Five human serum samples were diluted 1000-fold with phosphate buffer before being analyzed using the p53 biosensor. Furthermore, the accuracy of the biosensor was tested using the standard addition technique. The detection results of the prepared electrochemical immunosensor for p53 protein are summarized in Table B. Recoveries of five spiked human serum samples ranged from 98.41% to 109.32%, and these values were acceptable for real-sample analysis.

As summarized in Table A, the immunosensor was compared with reported studies. The constructed sensing platform was shown to have a low LOD and a wide linear analysis range. This developed biosensor can be used as an inexpensive tool for quick and early detection of the p53 protein biomarker.

## 4. Conclusion

A PThi-g-PGM conjugated polymer-coated disposable ITO electrode was used to create a simple and novel electrochemical biosensor for determining the p53 marker. The PThi-g-PGM conjugated polymer not only facilitated electron transfer in redox probes due to its conductive property but also increased the surface area of the electrode for anti-p53 antibody attachment. Thus, more target p53 proteins could interact with immobilized anti-p53. This fabricated biosensor operated in a broad concentration range from 0.05 pg/mL to 15 pg/mL. Additionally, it had a low LOD (15.9 fg/mL), excellent sensitivity (1.279 kΩ pg^−1^ mL.cm^2^) and acceptable recovery (98.41%–109.32%). The sensitivity, specificity, and reproducibility of the sensing platform were improved thanks to the modification of the ITO surface with the PThi-g-PGM polymer. Consequently, the fabricated immunosensor was a promising and viable method for p53 protein identification in clinical diagnostics.

## Supplementary information

The synthesis of polymer (PThi-g-PGM) is shown in Figure S1. The PThi-g-PGM polymer brushes were successfully prepared through a series of reactions (esterification, oxidative polymerization, and ATRP) (Figure S1). The thiophene compound was synthesized with an esterification reaction, which was used as a monomer for the thiophene macroinitiator [1]. The polythiophene macroinitiator was prepared by oxidative polymerization of a monomer compound using the oxidant ferric chloride (FeCl_3_) [2]. The PThi-g-PGM polymer brushes were prepared with an atom transfer radical polymerization technique in anisole [3]. The FTIR spectrum of glycidyl group-bearing polythiophene (PThi-g-PGM) is shown in Figure S2. The aliphatic C-H bond vibrations of polymer (PThi-g-PGM) were seen in 2995-2938 cm^−1^ [4].

The strong signal observed around 1722 cm^−1^ was attributed to the C=O stretching vibration of carbonyl groups of repeating glycidyl methacrylate monomers. The peaks monitored around 1252 and 1139 cm^−1^ were attributed to symmetric and asymmetric stretching vibration frequencies of the ester groups in polymers [5]. As well as the peaks at 905 and 845 cm^−1^ were attributed to characteristic signals of C-O-C in the epoxy group, which were located in glicidyl metacrylate (GMA) repeatable units of polymer [6,7]. The bands related to the thiophene moiety in the main polymer chain were observed at 537 cm^−1^ (C–S–C stretching) [8].

The chemical structure of glycidyl group-bearing polythiophene (PThi-g-PGM) was also examined via Raman spectral technique. Raman spectroscopy is a more commonly used spectral technique that has been supported by FTIR spectral results. Raman spectra of glycidyl group-bearing polythiophene were given in Figure S3. The peak at 1730 cm^−1^ was attributed to the C=O stretching vibration of carbonyl groups of repeating glycidyl methacrylate monomers in the polymer. The characteristic signals of epoxy groups in repeating glycidyl methacrylate units in polymer were seen at 909 and 852 cm^−1^ and confirmed the presence of an epoxy ring in the polymer. The bands related to the thiophene moiety in the main polymer chain were observed at 535 cm^−1^ (C–S–C).

Figure S1 Synthesis of glycidyl group-bearing polythiophene (PThi-g-PGM).

Figure S2 FTIR spectra of glycidyl group-bearing polythiophene (PThi-g-PGM).

Figure S3 Raman spectra of glycidyl group-bearing polythiophene (PThi-g-PGM).

**Table S:** The fitted EIS spectra results of electrode modification steps.

(A) Biosensor surface	R _ct_ (kΩ)
ITO/PThi-g-PGM	3.668
ITO/PThi-g-PGM/anti-p53	4.160
ITO/PThi-g-PGM/anti-p53/BSA	4.774
ITO/PThi-g-PGM/anti-p53/BSA/p53	5.878

References1

NeisesB
SteglichW

Simple method for the esterification of carboxylic acids
Angewandte Chemie International Edition in English
1978
17
7
522
524
10.1002/anie.197805221
2

LanziM
PaganinL
Costa-BizzarriP
Della-CasaC
FraleoniA

Facile synthesis of soluble multifunctional polyalkylthiophenes
Macromolecular Rapid Communications
2002
23
10–11
630
633
10.1002/1521-3927(20020701)23:10/11<630::AID-MARC630>3.0.CO;2-9
3

IaconoM
HeiseA

Stable poly(methacrylic acid) brush decorated silica nano-particles by arget atrp for bioconjugation
Polymers
2015
7
8
1427
1443
10.3390/polym7081427
4

EdmondsonS
HuckWTS

Controlled growth and subsequent chemical modification of poly(glycidyl methacrylate) brushes on silicon wafers
Journal of Material Chemistry
2004
14
4
730
734
10.1039/B312513K
5

AydınEB
AydınM
SezgintürkMK

A highly sensitive immunosensor based on ITO thin films covered by a new semi-conductive conjugated polymer for the determination of TNFα in human saliva and serum samples
Biosensors and Bioelectronics
2017
97
169
176
10.1016/j.bios.2017.05.056
28599176
6

OhJ
LeeJ
KooJC
ChoiHR
LeeY


Graphene oxide porous paper from amine-functionalized poly (glycidyl methacrylate)/graphene oxide core-shell microspheres
Journal of Material Chemistry
2010
20
41
9200
9204
10.1039/c0jm00107d
7

AydınEB
AydınM
SezgintürkMK

Ultrasensitive determination of cadherin-like protein 22 with a label-free electrochemical immunosensor using brush type poly(thiophene-g-glycidylmethacrylate) modified disposable ITO electrode
Talanta
2019
200
387
397
10.1016/j.talanta.2019.03.082
31036200
8

SahinE
CamurluP
ToppareL
MercoreVM
CiangaI


Conducting copolymers of thiophene functionalized polystyrenes with thiophene
Journal of Electroanalytical Chemistry
2005
579
2
189
197
10.1016/j.jelechem.2005.01.017


## Figures and Tables

**Scheme:**
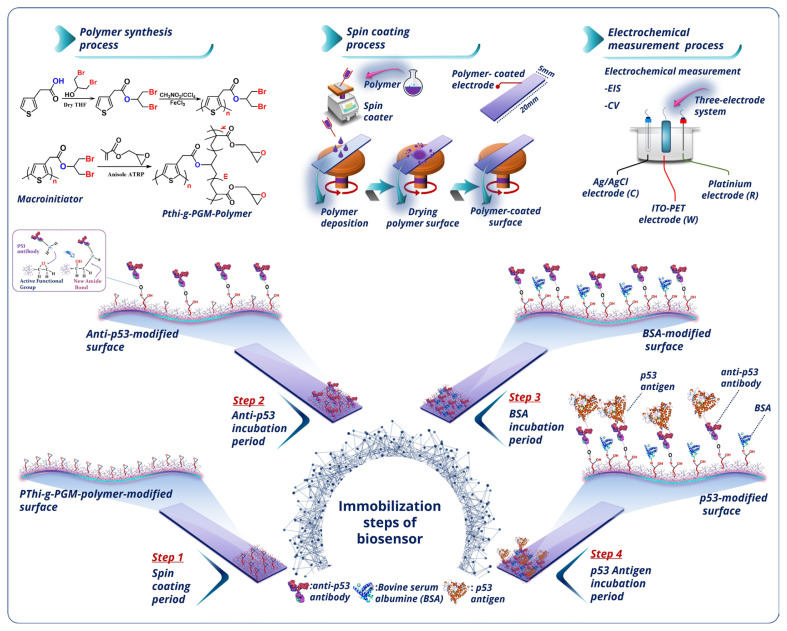
Fabrication process for p53 biosensor.

**Table:** Comparison of the performance of p53 biosensors and commercial kits (A) and detection of p53 protein in human serum samples using the designed immunosensor (B).

A) Protein p53 biosensors	Technique	Linear range	Detection limit	References
Silver nanoparticle-modified surface plasmon resonance	Optic	-	-	[10]
AuNP-modified screen-printed carbon electrodes	Electrochemical	2–50 nM	0.05 nM	[12]
DNA functionalized magnetic nanoparticle-modified fluorescent immunoassay	Optic	0.05–2 nM	0.008 nM	[13]
PEDOT: polystyrene sulfonate-modified glassy carbon electrode	Electrochemical	1–120 ng/mL	0.09 ng/mL	[34]
Graphene- and chitosan-modified screen-printed carbon electrodes	Electrochemical	0.2–10 ng mL	0.1 ng mL	[35]
Carboxylated NiFe_3_O_4_ nanoparticle- and polyethyleneimine-modified SPCE	Electrochemical	1–10^4^ pg/mL	5 fg/mL	[36]
p53 ELISA kit	Optic	0.23–15 ng/mL	65 pg/mL	Abcam
p53 ELISA kit	Optic	156–10,000 pg/mL	10 pg/mL	MyBiosource
p53 ELISA kit	Optic	0.3–80 ng/mL	0.3 ng/mL	RayBiotech
*PThi-g-PGM*-modified disposable ITO	Electrochemical	0.05–15 pg/mL	15.9 fg/mL	Current study

B) Sample	Measured by this system (pg/mL)	Added p53 (pg/mL)	Total amount after addition	% Recovery	% Relative difference
1	0.51	1.0	1.51	100.80	0.80
2	0.42	1.0	1.56	109.32	9.32
3	0.80	1.0	1.90	105.59	5.59
4	0.96	1.0	1.93	98.56	−1.44
5	1.52	1.0	2.48	98.41	−1.59
