# Supplementary figures and images for: Determination of p53 biomarker with a smart electrochemical biosensor based on brush polymer-functionalized disposable electrode
Source: Turk J Chem. 2025 Apr 4;49(3):371–81. doi: 10.55730/1300-0527.3736 (PMC12253966; doi:10.55730/1300-0527.3736)

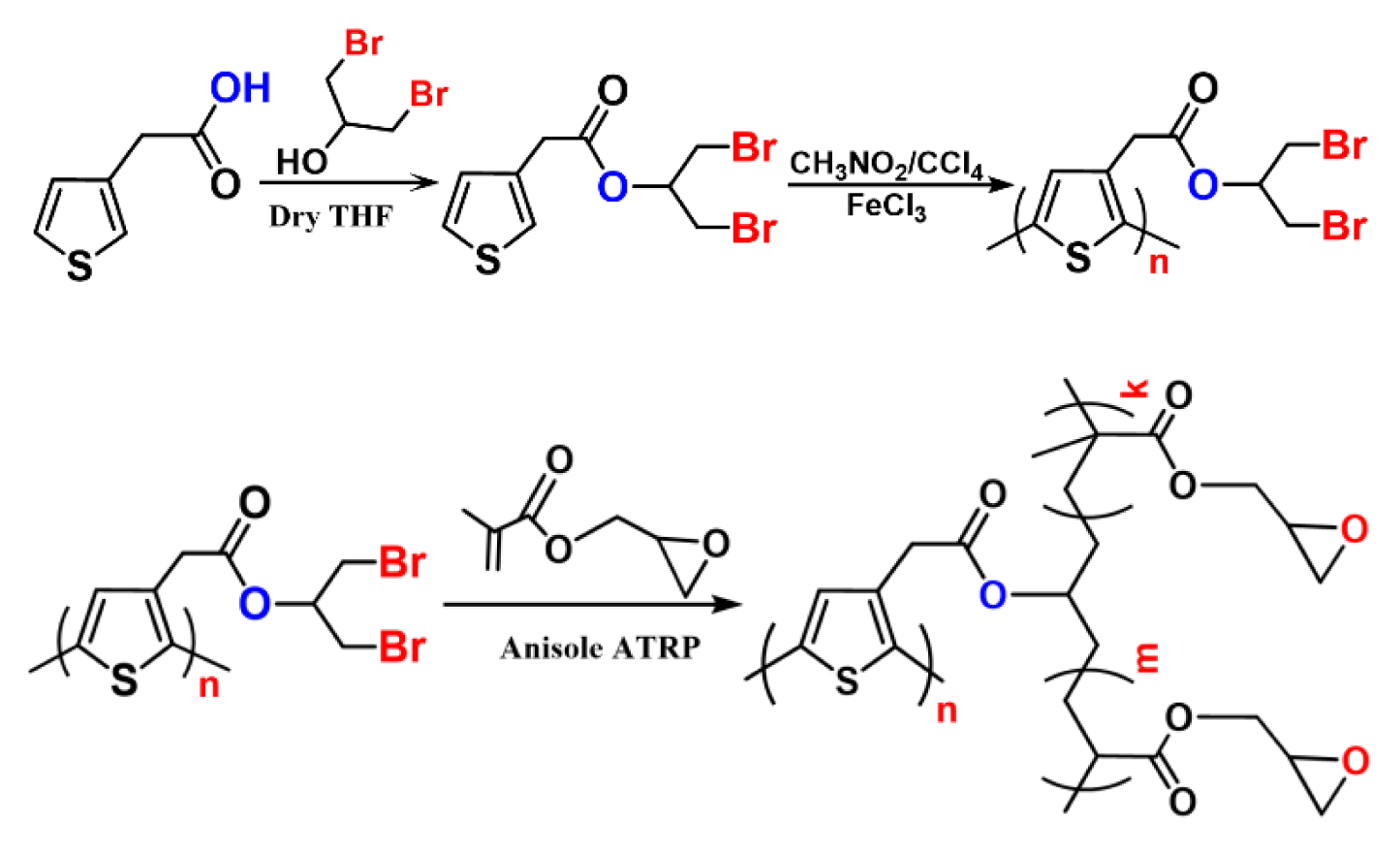

Supplement: Figure S1 — Synthesis of glycidyl group-bearing polythiophene (PThi-g-PGM). [file tjc-49-03-371s1.tif]

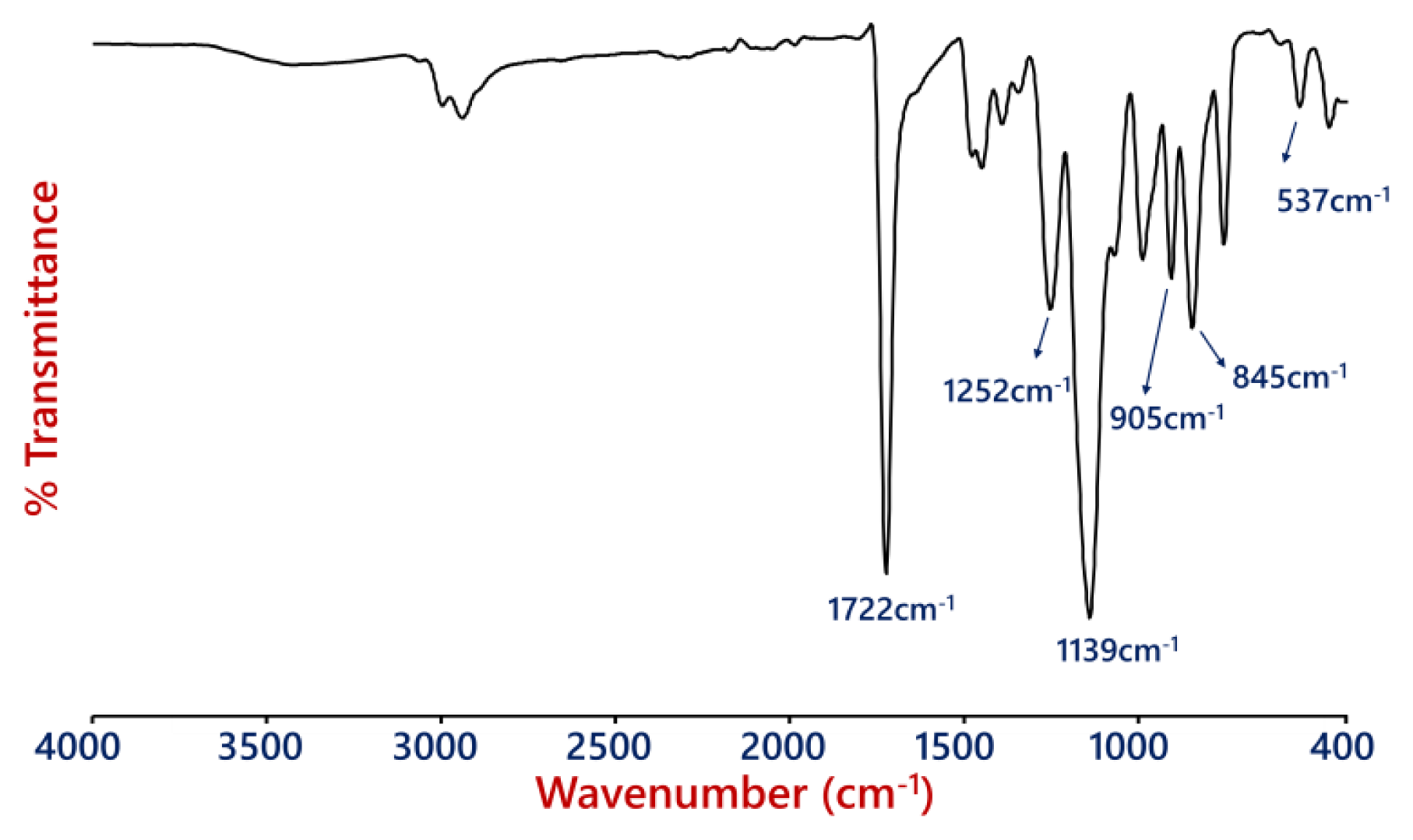

Supplement: Figure S2 — FTIR spectra of glycidyl group-bearing polythiophene (PThi-g-PGM). [file tjc-49-03-371s2.tif]

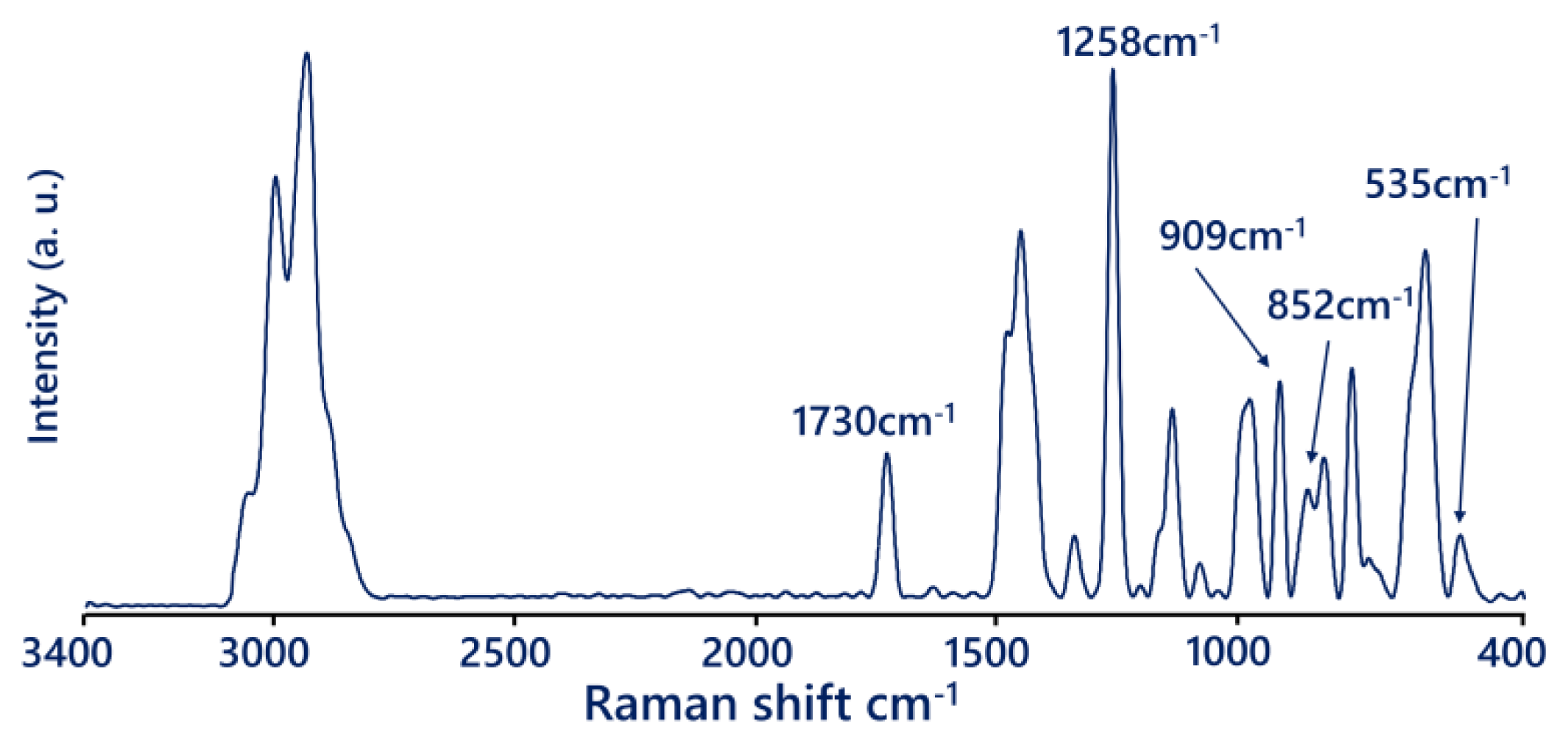

Supplement: Figure S3 — Raman spectra of glycidyl group-bearing polythiophene (PThi-g-PGM). [file tjc-49-03-371s3.tif]
